# Supplementary material for: The analgesic efficacy of pericapsular nerve group block in patients with intertrochanteric femur fracture: A randomized controlled trial
Source: PLoS One. 2022 Oct 13;17(10):e0275793. doi: 10.1371/journal.pone.0275793 (PMC9562218; doi:10.1371/journal.pone.0275793)
Supplement: Attachment — Submitted filename: Response to Reviewers.doc [file pone.0275793.s003.doc]

**Editor**

1. Please ensure that your manuscript meets PLOS ONE's style requirements, including those for file naming. The PLOS ONE style templates can be found at

https://journals.plos.org/plosone/s/file?id=wjVg/PLOSOne_formatting_sample_main_body.pdf and

https://journals.plos.org/plosone/s/file?id=ba62/PLOSOne_formatting_sample_title_authors_affiliations.pdf.

Respected Editors：thanks for your review , modifications have been made in the manuscript as requested.

2. In your Data Availability statement, you have not specified where the minimal data set underlying the results described in your manuscript can be found. PLOS defines a study's minimal data set as the underlying data used to reach the conclusions drawn in the manuscript and any additional data required to replicate the reported study findings in their entirety. All PLOS journals require that the minimal data set be made fully available. For more information about our data policy, please see http://journals.plos.org/plosone/s/data-availability.

"Upon re-submitting your revised manuscript, please upload your study’s minimal underlying data set as either Supporting Information files or to a stable, public repository and include the relevant URLs, DOIs, or accession numbers within your revised cover letter. For a list of acceptable repositories, please see http://journals.plos.org/plosone/s/data-availability#loc-recommended-repositories. Any potentially identifying patient information must be fully anonymized.

Important: If there are ethical or legal restrictions to sharing your data publicly, please explain these restrictions in detail. Please see our guidelines for more information on what we consider unacceptable restrictions to publicly sharing data: http://journals.plos.org/plosone/s/data-availability#loc-unacceptable-data-access-restrictions. Note that it is not acceptable for the authors to be the sole named individuals responsible for ensuring data access.

We will update your Data Availability statement to reflect the information you provide in your cover letter.

Respected Editors：we have uploaded a separate minimal data.

3. We note you have included a table to which you do not refer in the text of your manuscript. Please ensure that you refer to Table 4 in your text; if accepted, production will need this reference to link the reader to the Table.

Respected Editors：we have modified this and marked it in the manuscript.

4. Please include captions for your Supporting Information files at the end of your manuscript, and update any in-text citations to match accordingly. Please see our Supporting Information guidelines for more information: http://journals.plos.org/plosone/s/supporting-information.

Respected Editors：We have added the title of the supporting information file to the end of the manuscript.

5. Please review your reference list to ensure that it is complete and correct. If you have cited papers that have been retracted, please include the rationale for doing so in the manuscript text, or remove these references and replace them with relevant current references. Any changes to the reference list should be mentioned in the rebuttal letter that accompanies your revised manuscript. If you need to cite a retracted article, indicate the article’s retracted status in the References list and also include a citation and full reference for the retraction notice.

Respected Editors：We have checked the reference list and made corrections to ensure it is complete and correct.(all revisions have been marked in the manuscript)

**Reviewer 1**

1. At the end of introduction, can the authors state the primary and secondary objectives.

It has been supplemented in the introduction section of the manuscript.We will be happy to edit the text further, based on helpful comments from the reviewers.

2. Can the randomisation information include the allocation ratio.

It has been supplemented in the Randomization and Blinding section of the manuscript.We will be happy to edit the text further, based on helpful comments from the reviewers.

3. Can the authors define their population of analysis for the primary analysis (e.g intent to treat), secondary and safety.

It has been supplemented in the discussion section of the manuscript.We will be happy to edit the text further, based on helpful comments from the reviewers.

4. Table 1, as this this is an RCT, its recommended not to formally test baseline characteristics, any difference are simply due to chance and also do not serve a useful purpose so p-values should be omitted (see "Comparisons against baseline within randomised groups are often used and can be highly misleading by Bland JM, Altman 2011").

We thank the reviewer for pointing out thi issue.We studied the document you provided and found that there was a misunderstanding. The baseline written on the form was a clerical error and has been deleted in the text. In our research groups were be compared directly by two-sample methods.

5. Any correction taken into account for multiple testing, i.e testing in each timepoint, increase you chance of finding a significant result?

We thank the reviewer for pointing out thi issue. We have taken this situation into consideration,VAS score belongs to grade data and does not conform to the normal distribution, so we use the median (interquartile spacing) for statistical description, and use Kruskal Wallis H test to get the result P < 0.05. It can be considered that the distribution of scores in each group is not the same, and the difference is statistically significant, and then compare them at different time points to find out the positive results.

6. Can the authors state whether a statistical analysis plan was signed off prior to unblinding?

This study developed a statistical analysis plan before Unblinding. The staff who conducted the data analysis did not understand the processing methods of each group. They only selected the corresponding statistical analysis method according to the type of data collected, and then unblinded after completing the statistical analysis of each group of data.

7. Was there an oversight committee to monitor the safety of patients, its not mentioned in the manuscript?

The Ethics Committee supervised the implementation of this study and protected the rights and interests of patients. Relevant contents have been supplemented in the study design and subjects section of the manuscript.

**Reviewer 2**

Why do you think there was zero incidence of quadriceps motor weakness in the PENG block group compared to prior referenced studies that showed some degree of motor block, although still lower than fascia iliaca? You attributed it related to lower concentration or was the small sample size not adequately powered to detect secondary outcome measurements? Also would have been useful to measure quadriceps muscle strength at timepoints later than 6 hours.

We thank the reviewer for pointing out thi issue.We consider that this situation may be the reason for the concentration. The reference study nerve block used 0.5% ropivacaine, and this study used 0.375% ropivacaine.

In addition to the time point of 6 hours after operation, we also measured the quadriceps femoris muscle strength at 12 hours and 24 hours after operation, but there was no positive result, so it was not included in the result.

How was the method of passive exercise testing decided on? Is there a basis for using a 15 degree passive hip elevation?

We refer to reference 15 for the specific methods of passive motion testing.We will be happy to edit the text further, based on helpful comments from the reviewers.

How long after block performance were patients induced and surgery started? As t1 is 30 min post block time, does an increased length of time between block performance and starting the timer at the conclusion of surgery influence block duration when measured postop? The length of surgical time is shown but not the length of time between T1 and T2.

We thank the reviewer for pointing out thi issue.We made statistics on T1-T2 of patients in each group, and the results were not statistically significant. Table1 has been supplemented with relevant data.
